# Analysing reduced tillage practices within a bio-economic modelling framework

**DOI:** 10.1016/j.agsy.2016.04.005

**Published:** 2016-07

**Authors:** Toby J. Townsend, Stephen J. Ramsden, Paul Wilson

**Affiliations:** Division of Agricultural and Environmental Sciences, University of Nottingham, Sutton Bonington Campus, College Road, Sutton Bonington, Loughborough LE12 5RD, United Kingdom

**Keywords:** CAP, Common agricultural policy, CT, conventional tillage, DRT, deep reduced tillage, GHG, greenhouse gas, GM, gross margin, NE, net energy, NM, net margin, WOSR, oilseed rape, RT, reduced tillage, RP, rotational ploughing, SI, sustainable intensification, SRT1, shallow reduced tillage 1, SRT2, shallow reduced tillage 2, SB, spring barley, WB, winter barley, WFB, winter field beans, WW, winter wheat, ZT, zero tillage, Reduced tillage, Bio-economic modelling, Sustainable intensification

## Abstract

Sustainable intensification of agricultural production systems will require changes in farm practice. Within arable cropping systems, reducing the intensity of tillage practices (e.g. reduced tillage) potentially offers one such sustainable intensification approach. Previous researchers have tended to examine the impact of reduced tillage on specific factors such as yield or weed burden, whilst, by definition, sustainable intensification necessitates a system-based analysis approach. Drawing upon a bio-economic optimisation model, ‘MEETA’, we quantify trade-off implications between potential yield reductions, reduced cultivation costs and increased crop protection costs. We extend the MEETA model to quantify farm-level net margin, in addition to quantifying farm-level gross margin, net energy, and greenhouse gas emissions. For the lowest intensity tillage system, zero tillage, results demonstrate financial benefits over a conventional tillage system even when the zero tillage system includes yield penalties of 0–14.2% (across all crops). Average yield reductions from zero tillage literature range from 0 to 8.5%, demonstrating that reduced tillage offers a realistic and attainable sustainable intensification intervention, given the financial and environmental benefits, albeit that yield reductions will require more land to compensate for loss of calories produced, negating environmental benefits observed at farm-level. However, increasing uptake of reduced tillage from current levels will probably require policy intervention; an extension of the recent changes to the CAP (‘Greening’) provides an opportunity to do this.

## Highlights

• Reducing tillage intensity provides economic, energy and GHG emissions benefits.
• Using reduced tillage has implications for crop selection.
• Risks of yield penalties may explain why rotational ploughing is common.

## 1 Introduction

In the face of a growing world population, increased resource scarcity and the challenges of climate change mitigation, there is an increasing need for adaptation in agriculture and agricultural systems towards practices that lead to “Sustainable Intensification” (SI; Wilson, 2014). Within arable systems dominated by combinable crop production (e.g. wheat, oilseed rape), changes to cultivation practices, for example towards *reduced tillage*^1^ (RT), *conservation tillage* or *zero tillage* (ZT), have the potential to provide multiple environmental benefits (Holland, 2004) that would contribute towards SI objectives. These cultivation practices do not involve soil inversion (which occurs with ploughing); however the extent of soil disturbance typically ranges from intensive deep RT (e.g. tine harrows) to very minor soil disturbance in ZT (e.g. direct drilling).

RT provides benefits in areas prone to soil erosion including reduced soil erosion, pesticide runoff and watercourse sedimentation, improved soil quality, reduced leaching of nutrients and lower greenhouse gas (GHG) emissions (Fawcett and Towery, 2002, Holland, 2004, Morris et al., 2010). In humid temperate regions, such as northwest Europe, soil erosion is less of a problem and the environmental benefits of RT systems are less certain (Davies and Finney, 2002). RT systems have, however, been found to have lower GHG emissions and more favourable energy balances because of a reduction in machinery use (e.g. Knight, 2004). Reduced machinery use also leads to cost savings (Vozka, 2007), which is the primary driver of RT use in these areas (Davies and Finney, 2002). Studies have specifically identified that RT has lower fuel costs (e.g. Sijtsma et al., 1998, Šarauskis et al., 2014). Fewer machinery operations are also required with RT leading to reduced labour costs and improved timeliness of crop operations (Morris et al., 2010). When comparing RT with conventional tillage (CT) Verch et al. (2009) identified increased net returns from a German RT system of approximately €100 ha^− 1^.

Whilst clear financial benefits of RT practices have been observed, crop yield effects are less clear. Van den Putte et al. (2010), in reviewing Europe-wide field experiments, found an average yield reduction of 4.5% from RT (from 563 observations across different experimental years) though when ZT was considered individually there was an average yield penalty of 8.5% (171 observations). Arvidsson et al. (2014) found an average yield reduction of 1.8% from shallow RT experiments in Sweden (918 observations) and 9.8% lower for ZT (226 observations). Crop-specific effects of RT are confirmed by Van den Putte et al. (2010) with winter cereals and maize responding unfavourably to RT whilst the yields of other crops were unaffected. Climate-specific effects have been found, with a meta-analysis by Ogle et al. (2012) reporting reductions in yield for ZT systems for wheat and maize in the Northeast of the US, but increased yields in more southerly areas. Although RT tends to show an average yield reduction, when individual field experiments are considered, yields can be greater than with inversion-based tillage (e.g. Knight, 2004, Verch et al., 2009).

Although fuel, labour and machinery costs have been estimated to be lower for RT systems, there can be additional costs in RT systems resulting from greater weed, pest and disease burdens. Where present or where there is perceived to be a risk of their presence, farmers will apply additional crop protection inputs. Generally, extra herbicide is required for weed control under RT (Melander et al., 2013). Models of RT system costs have accounted for input use variability and have concluded that reduced fuel costs outweigh the costs of additional pesticide inputs (e.g. Lafond et al., 1993, Nail et al., 2007, Vozka, 2007). Greater amounts of fungicides may also be required, depending on the preceding crops in the rotation (Bürger et al., 2012). The fate of crop residues also influences tillage system costs as leaving crop residues in situ in RT systems can potentially increase molluscicide and fungicide requirements (Soane et al., 2012).

Consequently, whilst RT within a northwest European context provides possible cost and GHG savings, the potential trade-offs of RT approaches include yield reductions and increased crop protection costs. Currently, approximately 30–40% of arable land in England is under RT (Defra, 2010, Townsend et al., 2016). Given the identified benefits associated with the technique, it is pertinent to determine why there is not a greater area of land under RT.

Previous studies noted above have largely focused upon single issues of relevance to RT (e.g. profit; Verch et al., 2009); however, to achieve SI objectives it is necessary to examine the changes to cropping system approaches within a wider, system-based context. Sørensen et al. (2014) used a system-based approach to investigate tillage practices, demonstrating the value of this approach. This current study aims to address this issue, specifically utilising a bio-economic model, building upon Glithero et al. (2012), to investigate the influence of tillage type on a farm system and its outputs. Within our approach, we quantify the benefits, trade-offs and costs associated with different cultivation and crop establishment practices within a UK arable farm context.

## 2 Methodology

### 2.1 MEETA model

The MEETA (Managing Energy and Emissions Trade-Offs in Agriculture) model is a bio-economic optimisation model that determines optimal crop mix for three primary objectives: profit and net energy (NE) maximisation, and GHG emission minimisation. Profit is measured by total gross margin (GM), i.e. value of sales less variable costs of production for a given harvest year. Output from runs under each objective allows comparison of trade-offs between these competing objectives: for example, how much profit is foregone from reducing GHG emissions. The model was originally developed to establish trade-offs associated with increasing the supply of agricultural feedstocks for bioenergy production (Glithero et al., 2012). The model has also been used to consider the economic and environmental impacts of including dedicated energy crops (miscanthus and short rotation coppice grown for biofuel feedstock) within farm cropping systems and the extent to which marginal land is suited to bioenergy feedstock production (Glithero et al., 2015).

The model used here excludes dedicated energy crops and considers a 400 ha farm with a crop rotation that can include any of the following: winter wheat (WW), winter and spring barley (WB and SB, respectively), winter oilseed rape (WOSR) and winter field beans (WFB). The WW crop includes first, second and continuous wheats, i.e. first wheat is a wheat crop grown after a break crop (in the model this would be WOSR or WFB); second wheat is a wheat crop after first wheat and continuous wheat is where land is under wheat for three or more years. Straw can be baled from WB, SB and WW, or incorporated into the soil. Rotational constraints within the model limit the crops that can be grown, with break crops (WOSR and WFB) only being grown after a cereal crop. The crop mix generated is a single year representation of the average area of each crop grown.

A brief description of the three primary metrics of interest (GM, NE, GHG emissions) is given below; further details are provided in Glithero et al. (2012). The GMs include the variable costs of fertiliser, crop protection, seed, fuel for machinery operations and grain drying, and contractors' fees. Note that these GMs do not include the Basic Payment Scheme subsidy, part of the Common Agricultural Policy (CAP), as this is decoupled from production and therefore will not vary with crop mix. However, recent changes to the CAP (‘Greening’) do effect production and are included in the methods described below.

NE takes account of the energy required to produce the inputs, as well as the energy embedded in the machinery being used and the energy captured within the crop output. GHG emissions are calculated from the emissions required to produce fertilisers and sprays, the embedded emissions from machinery, soil N_2_O (nitrous oxide) emissions (calculated as 1.6% of applied nitrogen (N) released as N_2_O and a background soil emission of 1.4 kg N_2_O–N ha^− 1^ yr^− 1^). In reviewing the ZT literature, Soane et al. (2012) found that ZT tends to initially have higher N_2_O emissions but that this is not a consistent finding. Therefore, the emission level was initially kept constant for all tillage systems modelled. A sensitivity analysis was used to assess how important these assumptions are to overall GHG emissions for the different ZT systems considered below.

It was assumed that tillage practices do not influence fertiliser or crop protection requirements. Reducing tillage intensity has been suggested to alter fertiliser requirements. Some sources have found that greater N application is required during the first years of ZT and lower amounts in later years — in part because of reduced leaching (Soane et al., 2012); however, there is insufficient data to robustly consider this and, moreover, effects are likely to be highly site- and farm system-specific; they are, therefore, not included in the model.

The original model contains an intensive conventional tillage (CT) process consisting of a single pass of a plough followed by two passes of a power harrow. Work-rates for different machinery operations (ABC, 2011) are based on a heavy soil type and thus represent a relatively energy-intensive tillage system. The CT system used in the original model was modified to reflect a range of different RT systems. A number of scenarios were considered to provide a systems approach to determining the value of RT systems. These are listed below but more details are given in the further sections of the methodology.

• Baseline scenario: In this scenario, the model parameters and assumptions reflected market conditions in 2011, which is identical to those in the original study (Glithero et al., 2012). These prices were specifically maintained to allow a direct comparison to the outputs presented in the previous work, with the current work, without the conflating effect of introducing more recent prices. All model scenarios, excluding the price sensitivity scenario, are based on the 2011 market conditions.
• Net margin scenario: To capture tillage system impacts on farm finances, total farm net margin (NM) was calculated as GM less machinery costs (ownership and running costs) and labour costs. This was calculated for each RT system based on the optimised crop mix and associated machinery usage for profit maximisation.
• Yield penalty scenario: To consider yield impacts from RT systems, the trade-off points were determined where yield penalties negate any benefit for the ZT system over the CT system. This took into account any potential extra land required to maintain overall production levels and the GHG emissions associated with extra land. This was conducted for the ZT system as it is the most different to CT and the system most likely to suffer yield penalties.
• Weed control scenario: As RT systems, in particular ZT, are associated with an increased weed burden. The additional costs for herbicides were considered for the ZT system. The place of spring barley within the rotation was considered with respect to weed control through stale seedbeds.
• ‘Greening’ scenario: Rotational constraints were added to consider the impact of the ‘Greening’ requirement of the Basic Payment Scheme. This was conducted for the CT and ZT systems.
• Price sensitivity scenario: To assess how prices influence the RT systems, price sensitivity considered the crop mixes and outputs for market conditions in 2014. This included the ‘Greening’ rotational requirements.

### 2.2 Baseline scenario

RT systems in England employ a wide range of equipment and tillage practices; in particular, there is variation in tillage depth and number of passes (SMI, 2005). These vary with soil and weather conditions, crops and crop positions in rotations. To consider this level of variability, a number of different RT systems are compared within our approach (Table 1). These range in intensity from highly intensive in deep reduced tillage (DRT), to low intensity for two shallow reduced tillage practices (SRT1 and SRT2), to negligible soil impact for ZT. There is also a rotational ploughing (RP) system where both ploughing and SRT are used within the rotation.

Work-rates for DRT and SRT2 were taken directly from ABC (2011), whereas the work-rate for SRT1 was calculated from average contractors' work-rates. The number of cultivation passes is two for all crops and tillage options apart from DRT, wherein WFB only has a single pass (Table 2). The one-pass cultivator requires a large tractor, whereas the disc and tine harrows require a medium tractor. There is no secondary tillage and it is assumed that two passes provide a tilth suitable for establishment of the next crop. All tillage options include the same drill as the original model, apart from WOSR, which is assumed to be broadcast simultaneously with the tillage operations in all RT options except for ZT where the seed drill is required.^2^ The indirect energy and GHG emissions are calculated based on the weight of the equipment assuming that it is constructed of steel (Table 3).

### 2.3 Net margin scenario

The NM is similar to the adjusted GM used in Glithero et al. (2015) to investigate ownership of machinery; however, within the Glithero et al. (2015) study, depreciation was calculated irrespective of machinery usage rate. The NM used in this current analysis includes depreciation and labour costs, which were adjusted to reflect machinery usage, the inclusion of which provides a more realistic NM metric. This is important for the current study as overall machinery use will depend on the type of tillage operations used as well as the crop mix. Non-tillage and drilling costs, such as fertiliser application and pesticide spraying, were assumed to be unaffected by the type of tillage system employed.

Machinery purchase prices and labour costs were taken from ABC (2011), whereas depreciation rates, spares & repairs costs, and insurance rates were taken from ABC (2001). The depreciation rate was taken as a straight-line depreciation with the rate calculated based on the hours of machinery used; for example, the annual depreciation rate for a medium tractor ranges from 15% for a use of 500 h yr^− 1^ to 27% for a use of 1500 h yr^− 1^. Interest on capital was assumed to be 3%.

### 2.4 Yield penalty scenario

To investigate whether potential yield penalties from RT outweigh any potential cost saving benefits, sensitivity analysis was undertaken to determine acceptable threshold yield penalties. Whilst actual yield penalties for RT in England can be found in the literature, these frequently relate to data obtained prior to the ban of stubble burning in England and Wales (*The Crop Residues* (*Burning*) *Regulations 1993*); in these studies the straw was burnt prior to the next crop, which is likely to have aided weed and disease control. Yields from shallow RT are lower after straw incorporation rather than straw burning (Graham et al., 1986, Christian et al., 1999), suggesting RT systems are now less favourable following the straw burning ban. Other evidence suggests that WW yields tended to be lower under RT (Turley et al., 2003), whereas Knight (2004) found higher yields under RT. Hence, given this variability in data, the impact of yield reductions was examined by reducing yields for all crops from the 100% baseline to establish the threshold at which the benefits derived from reduced machinery use over the CT system are negated.

### 2.5 Weed control scenario

As RT systems tend to be associated with additional herbicide costs, this is considered in the model. In general, a multi-purpose herbicide can be applied prior to planting for control of weeds. However, where weeds have become established and have started developing herbicide resistance, more specialist herbicides are required. The most problematic weed in the UK is black-grass and there are a number of different herbicides commercially used to control it. Lutman et al. (2013) suggests that farmers are spending between £30 and £85 ha^− 1^ for herbicides to control black-grass. In the model a scenario was considered wherein black-grass herbicides are applied every other year at a cost of £85 ha^− 1^ based on the extreme value given by Lutman et al. to reflect the typically greater use of herbicides in RT systems.

One method for controlling weeds in cropping systems is through stale seedbeds and later-sown crops. The MEETA model includes the option for the farm to grow spring barley (SB); however, this was not selected by the original model because of its low yield. Sensitivity analysis was used to examine the level of yield penalty required (in other crops) before SB, without a yield penalty, was selected by the model.

### 2.6 ‘Greening’ scenario

Recent changes introduced as part of the Basic Payment Scheme of the EU's Common Agricultural Policy (CAP) mean that some of the original optimal crop mixes within the optimised MEETA model would not now be allowed. To gain the full payment, *Greening* criteria must be met. Where more than 30 ha of land is planted, at least three crops must be grown and the two main crops cannot cover more than 95% of the land (Defra, 2014a). Greening also requires that 5% of land is in Ecological Focus Areas (e.g. buffer strips around fields, hedges). The model assumes that the farm already meets these criteria as they are unlikely to interact with the cultivation method used by the farmer. Constraints were added to limit the two main crops to no more than 380 ha; thus at least three crops had to be grown. Note that the rotational constraints ensure that the model selects crops in proportions that are agronomically appropriate (e.g. first wheat can only follow a break crop).

### 2.7 Price sensitivity scenario

After running MEETA using the default (2011) price values to provide direct comparison with the results from Glithero et al. (2012), the model was run with prices updated to a recently observed lower commodity price market environment (2014). The rotational constraints of the ‘Greening’ scenario were maintained for this scenario. Diesel costs were assumed to be unchanged. Fertiliser prices were taken from (ABC, 2014): the 2014 N price of £750 t^− 1^ N is 20% lower and the 2014 phosphorous price of £620 t^− 1^ P_2_O_5_ is 55% greater than the default values. Potash price for 2014 was similar (2% lower) to that used in the original model. Chemical costs are the authors' own calculations based on the overall crop protection costs for crop types from ABC (2011) compared with ABC (2014; Table 4). Crop prices were generally lower in 2014 than in 2011 (Table 5).

NMs were also calculated for 2014 using recent machinery costs, work-rates and labour wages (£10.19 h^− 1^) from ABC (2014). Contractor fees were based on machinery costs assuming a ‘high’ usage rate, a 25% overhead and a 35% surcharge on the labour rate (£13.76 h^− 1^). Use of more recent data has a relatively small impact, although costs of processing straw (‘baling’) increase by circa 17% (Table 6).

## 3 Results

### 3.1 Baseline scenario

Replacing a plough-based CT system with RT changes the optimal crop mix when maximising profit and NE but for minimised GHG emissions the model maintains the same crop mix (Table 7). For optimised profit, introducing RT based on RP, the system favours increasing WOSR area (to half the rotation) whilst increasing WW slightly, at the expense of WB. WOSR is established after RT practices, whereas the WW and WB are established after ploughing under the RP scenario. Systems where all tillage is RT (i.e. no ploughing) favour increased areas of WW and WOSR over WB, reflecting the increase in available resources and different timings of operations for WB relative to WW and WOSR, with WB providing earlier harvesting than WW and thus spreading work load over a longer period.

For optimised NE, the RP and full RT systems (SRT1, SRT2, DRT and ZT) still favour half WW with straw harvested but WOSR is now favoured over WFB as the break crop; this is due to the WOSR crop being broadcast from the tillage machinery under these full RT systems, which avoids having a separate seed drilling operation (in the model, WFB is drilled). As with CT, the optimal crop mix for minimised GHG emissions for the RP and full RT systems has WW, with the minimum N fertiliser level and WFB in equal proportions.

For all tillage systems, GHG emissions vary substantially across the three objectives, largely in response to the amount of purchased N used; differences in profitability and NE are relatively small in comparison.

GMs were 14–25% greater for the RT systems compared with the CT system. The slow work-rate for the power harrow in CT results in time and labour requirements exceeding the available farm resources and, therefore, contractors are required to complete the process. The cost of the contractors adds approximately £85 ha^− 1^ onto the costs of the CT system compared with the full RT systems (Table 8). These are additional to the contractor fees incurred by all tillage systems for the use of the baler and swather.

Although the RP system only has 50% RT, the GMs were much greater than for the CT system: lower time and labour requirements for land preparation free up sufficient resources on farm to conduct these operations and, therefore, contractor requirements are much lower, significantly reducing costs. The full RT systems have similar GMs; interestingly, DRT has an almost identical GM to SRT1. Although SRT1 requires a smaller tractor, the work-rate is lower negating the benefit of using a lower-powered machinery input.

Net energy increased for the RT systems. For the ZT system, the optimised NE crop mix gives a NE value of 7.7% greater than that in the CT system. For the ZT system, optimising crop mix for maximising NE leads to only £11 ha^− 1^ (approximately 1%) foregone compared with the optimised crop mix for GMs.

When the crop mix was optimised to minimise the GHG metric, emissions were lower from the RT systems, with the ZT system having 16.4% lower emissions than the CT system. In the ZT system, optimising GHG emissions leads to a £174 ha^− 1^ (approximately 19%) reduction in profitability compared with the optimised crop mix for GMs. These result from lower fuel use and lower allocation of embedded energy and GHG emissions in the machinery.

Small changes in the N emission factor has large impacts on overall emissions because of the large impact of emissions from applied N. For the maximised GM scenario, increasing N emissions from 1.6% to approximately 2.5% negates any GHG emission reduction of ZT compared with CT with the standard N emission factor. This difference is less pronounced for the minimised GHG emission category as overall N fertiliser application is lower allowing the N emissions factor to be up to 3.8% before parity is reached with the CT system.

### 3.2 Net margin scenario

The RP system results in similar machinery costs to the CT systemeven though machinery use is reduced. Two sets of machinery are still required — RT machinery and a plough (Table 8). The full RT systems have greater NMs resulting from a combination of lower machinery, fuel and labour costs. The NM for the ZT system is £256 ha^− 1^ greater than that in the CT system. Although DRT has a similar GM to SRT1, the NM for SRT1 is £49 ha^− 1^ greater, reflecting the extra costs associated with DRT (a large tractor is required). These results indicate that greater financial benefits are derived from using RT than the GMs suggest. This is because NMs take into account the benefits of reduced labour and machinery costs alongside the reduced fuel use.

### 3.3 Yield penalty scenario

**Fig. 1:**
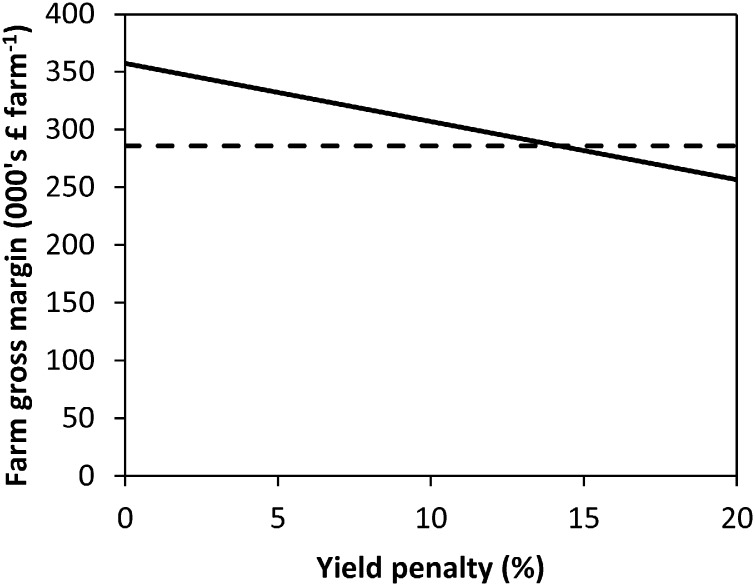
Gross margins per farm for the ZT system with crop yield penalties (solid line). The dashed line represents the GM per hectare for the CT system without yield penalties.

Sensitivity analysis with reduced crop yields for the ZT system (whilst leaving yields of the CT system unchanged) shows that an overall yield reduction of 14.2% is required for the GM of the ZT system to equate to that of the CT system (Fig. 1). The results presented in Fig. 1 were calculated by reducing crop yields within the model and re-optimising, repeating until the yield point is obtained, wherein financial parity with the results from the CT system (without yield penalties) occurs. The constant linear relationship observed within Fig. 1 derives from all variable costs being fixed regardless of yield except fuel costs for grain drying, which are directly proportional to grain yield.

**Fig. 2:**
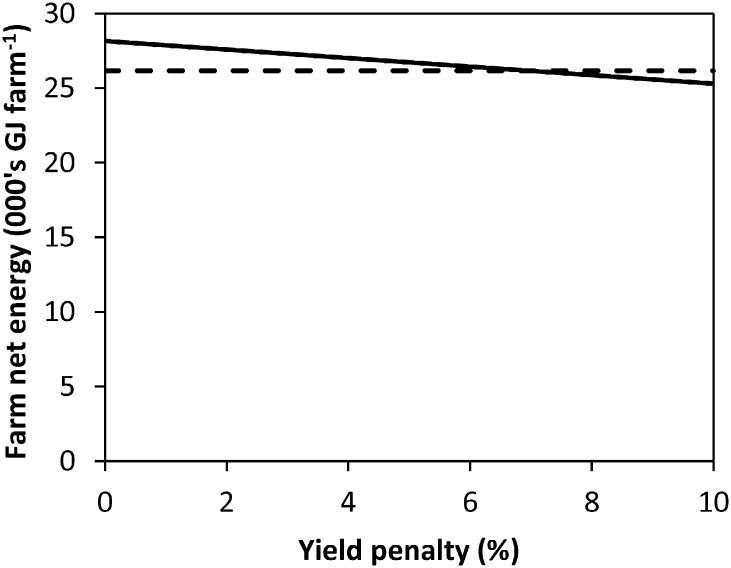
Net energy per farm for the ZT system with crop yield penalties (solid line). The dashed line represents the net energy for the CT system without yield penalties.

**Fig. 3:**
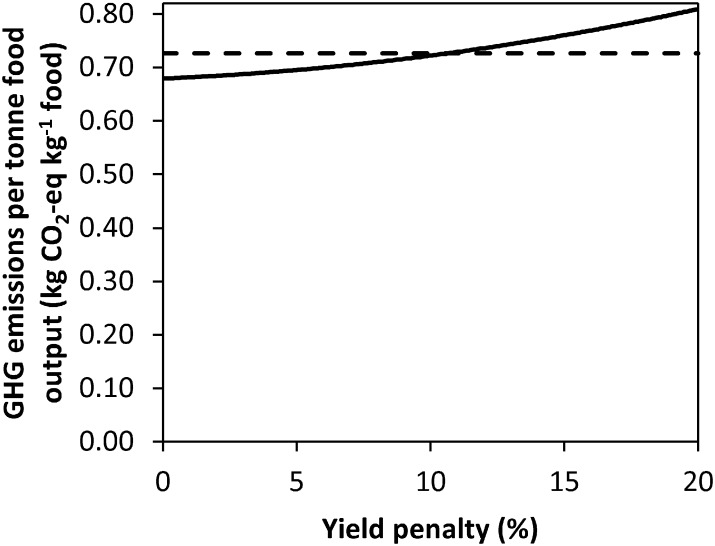
GHG emissions per tonne output per farm for the ZT system with crop yield penalties (solid line). The dashed line represents the GHG emissions per tonne output for the CT system without yield penalties.

An 8.1% yield reduction is required for the GMs of the RP to equal those of the CT system. With respect to the NE metric, the ZT system can incur a 7.0% yield reduction (Fig. 2) before NE is equal to the CT system; for RP the figure is 2.2%. GHG emissions were divided by the output of the system in kg of crop output; yields have to be 10.7% lower from the ZT system for the GHG emissions kg^− 1^ food output to be equal to those of the CT system (with normal yields; Fig. 3).

Considering the wider impacts of ZT, a yield reduction from ZT will require additional land to be used to compensate for the yield foregone. The indifference point where ZT becomes less economically beneficial than CT is observed at a yield penalty of 14.2%; given this yield reduction, approximately 24% more land would be required to maintain the same total food production in both tonnes of food and in terms of total calories.^3^ This disproportionate increase in additional land required results from the crop mix for ZT increasing the amounts of WW and WOSR within the optimal rotation, at the expense of WB, with WOSR having lower yields than WB. Given a yield penalty of 14.2% under the ZT system, maximised for total farm GM, GHG emissions for producing the same amount of food (and calories) as produced under the CT are greater in the ZT system than those in CT, negating the climate change benefits observed from ZT at farm level. Specifically, the total GHG emissions indifference yield point occurs at a 12.8% yield reduction; any yield penalty greater than 12.8% negates the farm level GHG emission savings of ZT.

### 3.4 Weed control scenario

GMs remain higher for RT systems even when applying additional herbicides to combat black-grass. In the extreme scenario of applying £85 ha^− 1^ for all crops, ZT still has higher GMs than CT, although these are slightly lower than the RP system (with no additional herbicides). For the calculation of NMs, the requirement for extra spraying adds an additional £4.81 ha^− 1^ to costs from machinery, fuel and labour. Black-grass control is unlikely to require such high frequency of application suggesting that RT remains profitable over CT even when there are weed problems.

One method of controlling weeds is delaying drilling (usually using a spring-sown crop) to allow time for a stale-seedbed. In the model SB was given as an option but is not chosen under standard conditions because its yields are much lower than the autumn-sown crops. For the ZT system, SB is only selected when the yield for all other crops is reduced by over 17% and even then, SB is only introduced on a small amount of land. To have a significant amount of SB within the rotation, a yield reduction of over 20% for all crops other than SB is required (Table 9). When the ZT system has SB as the cereal within the rotation, the GM of the ZT system is actually lower than that of the CT system when growing WB and WW.

### 3.5 ‘Greening’ scenario

The crop mix for the CT and RT systems optimising for GMs is unchanged but the optimised crop mix for maximising NE and minimising GHG emissions requires bringing in an additional crop to meet the policy requirements (Table 10). Overall, the financial changes are small. When optimising for minimum GHG emissions, this is the only scenario to grow SB, probably selected because of its lower N requirements. Under both the CT and RT systems the Greening requirement leads to changes in the crop mix for the maximised NE and minimised GHG emission scenarios.

### 3.6 Price sensitivity scenario

GMs were approximately 18–25% lower under 2014 market conditions as compared with the baseline scenario due to the general decrease in crop prices and an increase in chemical input prices, as well as the ‘Greening’ constraints (Table 11). For the CT system under 2014 market conditions, WFB was favoured over WOSR as the break crop due to the lower price for WOSR and a higher price for WFB. Under these prices, the crop mix for the full RT systems changes to 45% WW (with straw baled) and 50% WFB, with 5% WB to fulfil the ‘Greening’ requirements. Interestingly, the optimised crop mix for maximum profit is the same crop mix for maximum NE and it is also very similar to the crop mix for minimum GHG emissions when both are under the ‘Greening’ constraints; the only difference for the latter is that straw is not collected and the minimum N fertiliser rate is applied to WW.

The RT systems bring in WW to 50% of the rotation whilst replacing WB with WOSR as the third crop because of less pressure on resources. Whereas RP, DRT and ZT have only 20 ha for WOSR, the SRT systems have a greater amount of WOSR; this is due to an equal number of tillage passes for WFB as WOSR whereas for other tillage systems, WFB has fewer tillage passes, providing a benefit for WFB. Constraining WOSR to only 20 ha to force the same crop mix only reduces GM by £80 at the farm-level suggesting minor changes in tillage requirements can shift crop mix quite a lot. Having extra WOSR results in there being higher GHG emissions for the SRT and RP systems compared with CT as WOSR requires N fertilisers whereas WFB does not. For profit maximisation with the 2014 market conditions, the GM for ZT is approximately 19% higher than CT, whereas under the 2011 market conditions, the GM of ZT was 25% higher than CT.

## 4 Discussion

### 4.1 Metrics of sustainable intensification

By reducing tillage intensity farmers can increase their GMs and NE per hectare whilst lowering GHG emissions. Previous authors have identified crop yield reductions from RT systems in comparison to CT systems yet the benefits of RT systems are still observed when taking into account potential yield reductions. These yield reductions cited in the literature have typically been modest (less than 4.5% in magnitude for SRT as reported by Van den Putte et al., 2010); however, the yield penalties tend to increase with decreasing tillage intensity, so for ZT systems yield penalties can be higher. By contrast, the yield threshold testing approach presented here has identified that more substantial yield penalties from RT can be incurred, whilst still achieving a greater financial return than CT systems. Where RT systems lead to additional crop protection inputs, in particular for control of weeds such as black-grass, model results indicate that increased crop protection costs are not a large barrier to the financial viability of RT systems. This is in line with other studies (e.g. Vozka, 2007).

Reducing tillage intensity lowers fuel use resulting in increased NE and GM outputs, and decreased GHG emissions compared with CT. Fuel use reductions ranged from 23% for RP to 58% for the ZT system, which is towards the maximum fuel savings suggested by the SoCo Project Team (2009) in their review of the literature. The increased NE and decreased GHG emissions also result from reduced machinery use of the RT systems modelled and lead to lower embedded energy within machinery held on farm and hence lower emissions allocated to the farm systems.

Cost savings from the RT systems result from lower fuel inputs, lower contractor requirements, as well as the flexibility to grow a greater area of more valuable crops in the rotation, thus providing additional revenue. For CT, time and labour constraints during the soil cultivation period force the crop mix to include a third of the land as WB, a less valuable crop, to spread the workload and limit contractors' costs. As the utilisation of RT reduces cultivation time, a greater area of the more valuable crops, WW and WOSR, can be grown instead.

Cost savings represent a key driver of RT uptake in Northern Europe (Morris et al., 2010) and our results demonstrate substantial cost savings from the adoption of RT systems. In terms of current RT use, in England RT practices cover approximately 30–40% of arable land but these tend to be used in conjunction with ploughing. Townsend et al. (2016) defined mixed tillage systems as using both ploughing and RT practices within the rotation. These range from the use of ploughing at a specific place within the rotation (i.e. rotational ploughing, such as using it for the non-break crops in the current model) through to using it dynamically in response to specific conditions (i.e. strategic tillage). Only a small proportion of farms solely used RT and very few farms used ZT.

RP is commonly used because it allows farmers to gain some benefits from using RT whilst minimising the risk of yield penalties. In the current model RP had a more favourable GM than the reduction in fuel costs would suggest as the reduced machinery requirement meant that operations could be completed without exceeding farm resources, thus avoiding the need to use contractors for cultivation. This demonstrates that a partial reduction in ploughing can have relatively large financial benefits.

The model results show clear benefits of using RT over CT but there were only relatively minor differences in the GMs of the different full RT practices. The NMs show that greater benefits are found with lower intensity RT, demonstrating that NMs are a better means of establishing the value from changes to cultivation practices as they provide a more holistic financial metric. Even so, the financial benefits of ZT over shallow RT are relatively minor, even without taking account of potential yield penalties. Verch et al. (2009), in conducting a field experiment comparing tillage systems, found that in 17 out of 20 comparisons, financial returns did not significantly differ between ZT and SRT systems. The ZT treatment did suffer yield penalties resulting in the lower profitability; the authors suggest that over a longer time period the yield gap between SRT and ZT treatments would decrease, giving ZT a better profitability. Vozka (2007) found that ZT had a similar cost to SRT when ZT incurred additional herbicide costs. The model results show that a financial incentive to move away from plough-based agriculture exists, but that additional financial benefits from reducing tillage intensity further are fairly limited. This helps to explain why RT practices tend to remain quite intensive (based on the average depth of RT practices; Townsend et al., 2016) and ZT is relatively rare. Davies and Finney (2002) suggested that the risks of yield losses would also restrict the use of ZT and SRT.

Our results demonstrate that input and output price variability can lead to contrasting financially optimal crop mixes. For example, the increase in GM for ZT over CT were 25% in the original model but 19% with the lower 2014 prices; this is due to the optimal crop mix with lower prices favouring WFB, which has lower tillage requirements under the CT scenario and, therefore, the cost savings from moving to a RT system are lower than a rotation favouring WOSR. Together with the farm-specific factors, this strongly suggests that specific conditions on the farm, and with respect to market prices, could determine the extent of benefits from moving to a RT system from CT.

The model only captured one metric of sustainability (GHG emissions). RT practices are associated with other environmental benefits that were not captured by the model such as reducing water pollution resulting from leaching and run-off of pesticides, nutrients and soil sediment (Holland, 2004). There is also considerable uncertainty regarding the environmental benefits of RT as these tend to be very site-specific with a strong influence from factors such as climate, soil-type and topography. Capturing these for a model like MEETA is not possible based on the limited data that is available, and is beyond the scope of this current study.

As shown by Gibbons et al. (2006), uncertainty is also present in GHG emission estimates. As noted in our methodology here, the emissions of N_2_O are highly variable but tend to be initially higher for ZT systems. As the model results are sensitive to the N_2_O emission factor, this could mean the GHG emission savings are over-estimated. In contrast, greater emissions savings for ZT have been suggested through increased carbon (C) sequestration in the soil (Powlson et al., 2012). Sørensen et al. (2014) included changes in soil C in their calculation of net changes in GHG emissions resulting from switching to RT and this accounted for larger GHG emission reductions than from reduced machinery use. In the literature there is considerable uncertainty regarding this and it is disputed whether there is actually an increase in C from switching from CT to ZT; although soil C increases in the upper layers of soil, there is a decrease deeper in the soil, and thus ZT may not lead to greater C sequestration (Baker et al., 2007, Soane et al., 2012).

The SI concept is broadly based upon increased food production whilst impacts on the environment are either held constant or reduced. If RT practices lead to a yield penalty, would these practices still count as SI? The benefit of the system-based approach taken in the MEETA model is that it highlights that although the model can financially support a set amount of yield loss, leading to reduced GHG emissions at the farm-level, the wider GHG emissions when examined as a function of total food or calorie production are actually negated given this loss of yield. Hence, the corollary of farm-level SI practices derived from the implementation of RT maybe negative environmental outcomes globally. Specifically, given an approximate 13% yield reduction under ZT, local GHG emissions would be reduced, whilst globally, there would be an increase in GHG emissions to maintain total calorie production.

Even in the absence of a yield penalty, introducing RT leads to lower overall food production because RT practices reduce pressure on resources allowing the amount of WOSR to be increased in the crop mix, resulting in lower overall calorie production from the farm. This highlights that under free market conditions, farmers respond to price signals to maximise net financial return and as a consequence do not necessarily maximise overall food production. Within the framework presented in this study, the model assumes that farmers operate in a global market, and that their individual crop mix choice does not influence the market price.

### 4.2 Policy and practice

The change in the optimal crop mix demonstrates potential opportunities to change crop rotations when switching from CT to RT because of quicker field preparation. This has important implications for land-use management. In the model this has resulted in a less diverse crop mix in terms of a much smaller area of WB. Crop rotations are important to reduce pests and disease whilst maintaining or increasing soil productivity (Martin et al., 2006). The ‘Greening’ requirement discourages the growing of monocultures by requiring at least three crops to be grown to claim the full Basic Payment Scheme subsidy (Defra, 2014a); however, in practice, as we have modelled, a large proportion of the area can be in two crops: for example, the optimal crop mix under the maximum GM scenario would still be allowed under the ‘Greening’ requirements even though only a small area of WB is grown. The optimal crop mixes for maximising NE and minimising GHG emissions would not be allowed as there are only two crops; however, meeting the requirements would only have a marginal effect as only 20 ha (5%) would need to be replaced with an alternative crop.

Although RT provides extra flexibility in rotations for farmers by freeing up resources, it typically restricts the use of root crops, which are less suitable for RT practices. A standard risk management strategy that farmers adopt to address price and yield variability is to have a greater number of crops in their rotation (Hardaker et al., 2004); if RT systems reduce the flexibility of crop choices they will be less attractive to risk averse farmers. Utilising a mixed tillage system would provide farmers with the widest range of crops by reducing resource pressure whilst allowing tillage practices to optimally fit each crop grown. Townsend et al. (2016) found that the use of RT varied greatly with crop type, being extensively used for WOSR but only very rarely for root crops (potatoes, sugar beet) and field beans. The extent of RT usage thus partly depends on the crops grown.

Control of black-grass represents a major current and potential threat to arable cropping systems in the UK. Although the costs of herbicides to enable RT are not prohibitive to the use of RT, it may be that an additional herbicide application is insufficient to control weeds such as black-grass, especially when the weed burden has established itself. Ploughing is one method of controlling black-grass and the model considered the use of a mixed tillage system (i.e. RP). The results show that it is possible to incorporate RT with inversion, plough-based cultivations, and still achieve environmental and financial benefits.

However, for continuous RT, alternate strategies are required. There is evidence to support the use of stale-seedbeds using later-sown crops to reduce black-grass (Lutman et al., 2013). The quicker cultivation of land leaves a potentially larger time window in the autumn for a stale seedbed prior to autumn-sown crops, although delayed sowing can reduce yield (Hay and Walker, 1989). Inclusion of spring-sown crops within the rotation would allow a considerably longer period of stale seedbed and more effective means of controlling weeds (Chauvel et al., 2009). UK crop rotations tend to be dominated by WW and WOSR but increasing rotation length and diversity, such as by the inclusion of spring-sown crops, would reduce reliance on herbicides (Ferguson and Evans, 2010). However, incorporating other crops into the rotation could reduce profitability. In particular, spring-sown crops are generally financially unattractive to farmers (Pardo et al., 2010) and in cereal rotations are less common than autumn-sown crops. There is very little spring-sown wheat or oilseed rape in the UK and although there is more spring-sown barley than winter-sown barley, winter-sown barley tends to dominate in the south and east whilst spring-sown barley tends to dominate in the west and north (ABC, 2014, Defra, 2014b). This observed cropping pattern is reflected in the current model which does not select spring-sown crops, even when autumn sown crops necessitate bringing in contractors to prepare land for autumn-sown crops. Inclusion of SB in the ZT system negated the cost savings from lowering tillage intensity. With a potential reduction in the efficacy of herbicides and stricter legislation on use of crop protection chemicals, practices such as more diverse rotations and stale seedbeds may assume greater importance in the battle to control weeds.

An important caveat for the model results is that the benefits of RT depend on the baseline conditions from which measurement of these tillage changes takes place. The model considered quite an intensive CT system and the soil was “heavy”, giving slower work-rates. Thus, there was pressure on farm resources necessitating the use of contractors. Where time and labour is less constrained during the cultivation period less benefit is likely to be seen from utilising RT. The CT system in the model could forego the power harrow and use a ‘soil packer’ instead to break up soil clods, which would reduce costs whilst still maintaining ploughing as an option; this might be seen as more attractive to a farmer than bringing in RT, although this will depend on the relative performance of crops under each system. Soil type has an influence on the feasibility of RT (Davies and Finney, 2002, Morris et al., 2010) but also on the relative benefits where heavy soils, which have lower work-rates, have relative greater fuel savings (SoCo Project Team, 2009).

Given the possible environmental benefits of RT and the financial benefits previously noted, it is worth exploring the reasons why more land is not currently under RT. Townsend et al. (2016) presented some of the barriers preventing greater use of RT and some of these have been demonstrated in the MEETA model results.

There are a multitude of factors influencing the adoption of soil conservation practises in agriculture (Knowler and Bradshaw, 2007, Prager and Posthumus, 2010). Studies show inconsistency in the factors they identify as influencing adoption, which is partly due to factors tending to be site-specific and partly because of the variety of methodologies used to make assessments (Knowler and Bradshaw, 2007) — behaviour regarding adoption has been determined by personal, socio-cultural, economic, institutional and environmental approaches. Despite this variety, financial benefits are frequently identified as the key driver of whether or not soil conservation practices are adopted (Prager and Posthumus, 2010).

### 4.3 Machinery ownership

Machinery costs influence adoption of RT practices. When moving from CT to mixed tillage the requirement to hold machinery suitable for both CT and RT simultaneously will typically lead to greater machinery depreciation costs than would result from a single tillage system approach. This is demonstrated in the similar machinery costs between the CT and the RP systems. It is possible to have a RP system without the expense of additional equipment as farmers can directly broadcast seed of crops such as WOSR into the stubble of the previous crop, thus avoiding the expense of two sets of tillage equipment. Results from the MEETA model presented in Glithero et al. (2015) identified that decision making with respect to levels of machinery ownership was a key financial driver in determining optimal crop mix. In the MEETA model results, the SRT and ZT systems did not require the large tractor, which is partly responsible for the RT systems having much higher NMs. In contrast, the DRT system requires a powerful tractor, which accounts for the DRT system having lower NMs than SRT1, even though these two systems produce identical GMs. A key point here is that gaining the full benefits of switching from a CT system to a RT system would require a change in the machinery present on the farm: this is not a cost free process and includes both capital and learning costs. In the MEETA model the learning costs were not covered by the NMs but could have included a slower work-rate initially. As mentioned in Townsend et al. (2016) financial grants to facilitate the transition between CT and RT systems to cover these costs may be required to encourage further uptake. However, the benefits are uncertain and thus a farmer's attitude to this risk is also relevant. Farm size is a determinant of whether RT practices are likely to be used (Townsend et al., 2016) and this could be due to larger farms being able to finance much larger tractors and afford multiple sets of tillage equipment when mixed tillage systems are used.

### 4.4 Perceptions and penalties

Despite the potential financial, energy and environmental benefits of RT, uptake of these practices will be affected by the prior beliefs of farmers (Andrews et al., 2013), in particular regarding the yield penalties that may be incurred. The model does not capture specific risks associated with RT practices, such as severe weed problems. As noted, farmers tend to be risk-averse and it is possible that some farmers are continuing to use CT as there are strategic risks associated with switching to RT systems. These risks include yield penalties and weed problems but also extend to the problem of adopting a new technology that may not be suitable for a farmer's cropping system. The farmer's subjective probability of the effect of moving from CT to RT will also affect uptake with the assumptions about increased risks. These may be based on experience; RT was common in the UK in the 1970s but its use declined due to difficulties with increased weed burden (Davies and Finney, 2002). If farmers had experienced this they may be unwilling to try RT again, even though better equipment (e.g. seed drills) is available and there is greater knowledge of best practices.

Farmers' reluctance to adopt of RT based upon concerns about yield penalties are understandable given the paucity of data on yield impacts from the use of RT, in particular after the ban on burning stubble. On average, evidence suggests that yields are slightly lower under RT and this might discourage farmers from adopting RT. Yet under certain conditions there is the potential for RT systems to result in greater yields (Knight, 2004, Verch et al., 2009). One aspect potentially not accounted for in field experiments that compare RT systems is the commercial potential to drill crops earlier, as RT practices require less field preparation time per hectare than CT. The improved timeliness of field operations resulting from the lower labour and machinery requirements of the system could lead to better yields. Determining how yield reacts to certain soil types, climate, timeliness of crop establishment and cropping systems would provide better information for farmers to assist them in decisions regarding RT; however, as argued by Davies and Finney (2002) long-term yield experiments are expensive, time-consuming and particularly site-specific. Considering the results of the current study, RT systems generate greater GMs and NMs even when accounting for yield penalties and this suggests farmers should not focus on potential yield reductions but also consider cost savings, and hence margins, when making decisions about tillage practices.

Adoption of RT is also limited by farm-specific factors such as farm size, crop rotation, machinery available, climate, soil type and weed burden. As shown by Ogle et al. (2012), in the US, RT can have beneficial yield effects in drier, warmer climate conditions and is thus more suited to areas with these conditions. In the UK, where water-stress is less common, there is likely to be less incentive to adopt RT.

### 4.5 The future for reduced tillage

In combination, the above factors may go some way to explaining why only 30–40% of arable land in England is currently under RT. It is also possible that farmers are identifying that there are greater risks associated with low intensity RT (SRT and ZT) and are either using deeper reduced tillage or, where they are using SRT or ZT, also using rotational ploughing.

Although there is some financial encouragement for soil conservation practices in agriculture through the CAP, in general farmers are not rewarded for the positive externalities associated with the adoption of these practices. It has been suggested that farmers should be incentivised to use RT. For example, reducing tillage intensity has been suggested as a way of sequestering carbon in the soil (Powlson et al., 2012) and providing carbon credits for farmers using RT practices would encourage uptake (Alexander et al., 2015). However, there is no robust evidence that RT leads to increased soil C stocks; furthermore, as discussed above, there is still much uncertainty generally over the effectiveness of different interventions on greenhouse gas emissions.

This uncertainty extends to farmers' perceptions of RT: it has been said that UK farmers “regard soil conservation practices with suspicion as they perceive a great uncertainty on their effectiveness and impact on farm productivity” (Prager and Posthumus, 2010). This would seem to be a rational response given current levels of understanding. Davies and Finney (2002) emphasise the variability in the soil impacts found in RT field experiments and the uncertainty regarding the scaling-up of these impacts from field experiment to farm level. Townsend et al. (2016) question whether the current RT practices (deep tillage used in mixed tillage systems) provide the environmental benefits presented in the literature.

In reality, RT is part of a suite of soil conservation practices that could be used to improve the sustainability of agriculture. Alongside longer rotations and permanent soil cover RT is referred to as conservation agriculture and these practices complement each other and enhance the benefits derived from each practice individually. Incentivising the uptake of various components of conservation agriculture, such as longer rotations, could be operationalised through modified ‘Greening’ requirements and better soil practices implemented through the Soil Protection Review. Such practices would, on the evidence presented herein, represent a positive step towards increasing the sustainable intensification of agricultural system, enhancing the financial, environmental and energy outcomes from primary food production systems.

## 5 Conclusions

Reducing tillage intensity increased farm-level gross margins and net energy potential whilst lowering greenhouse gas emissions. Modelling to include flexibility in labour and machinery gave greater financial benefits as measured by net margin and lowers emissions still further. Given the relatively large threshold yield reductions that are required before RT systems are less financially attractive at the farm level than CT, this suggests that RT is one route towards sustainable intensification (SI). However, given that yield reductions will require an increased use of land to compensate for these yield penalties, the locally observed environmental benefits from RT may be negated when examined globally. The modelling framework within which these results were generated also allows us to quantify the farm-level impact of financial, energy and environmental metrics associated with this potential SI practice. Reduced tillage both increases and reduces crop choice flexibility; flexibility is increased by reducing work-rates for field preparation but reduced by preventing the growing of crops unsuited to RT. Mixed tillage systems offer the greatest flexibility, but may compromise some of the environmental benefits. Despite the potential financial benefits, uptake of RT is still relatively low at the time of writing, at 30–40% of the arable land area of England. There are a range of reasons that can explain this, including farmers' risk attitudes and perceptions and the benefits, particularly for weed control, of systems that include some ploughing. With better quantification of the GHG benefits and increased emphasis on ‘Greening’ the CAP, there is considerable scope for reconfiguring existing policy mechanisms to encourage greater uptake of a range of RT approaches.

## Figures and Tables

**Table 1:** Tillage systems investigated in the MEETA model in decreasing levels of intensity.

Tillage system	Abbreviation	Description
Rotational ploughing	RP	Reduced tillage (two passes of a medium disc harrow) for break crops but CT before wheat and barley
Deep reduced tillage	DRT	A one-pass cultivator, consisting of tines and discs. As the soil is heavy, we assume two passes.
Shallow reduced tillage 1	SRT1	Two passes of a medium disc harrow
Shallow reduced tillage 2	SRT2	Two passes of a spring-tine harrow
Zero tillage	ZT	Seed planted into the stubble from the previous crop

**Table 2:** Work-rates presented as the time in minutes required for a single pass over a hectare and number of passes required per crop by tillage system.

Tillage system	Field operation	Work-rate (min ha^− 1^)	Number of passes per crop				
WW	WOSR	WB	SB	WFB
CT	Plough (6 furrow; heavy land)	70	1	1	1	1	1
Power harrow 4 m; heavy land)	67	2	2	2	1	0
Precision drill	43	1	1	1	1	1
RP	Plough (6 furrow; heavy land)	70	1	0	1	1	0
Power harrow 4 m; heavy land)	67	2	0	2	1	0
Medium disc (2–3 m)	42	0	2	0	0	2
Precision drill	43	1	0	1	1	1
DRT	One-pass cultivator (4.5 m; heavy land)	24	2	2	2	2	1
Precision drill	43	1	0	1	1	1
SRT1	Medium disc (2–3 m)	42	2	2	2	2	2
Precision drill	43	1	0	1	1	1
SRT2	Spring-tine harrow (6 m; heavy land)	23	2	2	2	2	2
Precision drill	43	1	0	1	1	1
ZT	Precision drill	43	1	1	1	1	1

**Table 3:** Embedded energy and GHG emissions for the RT machinery. The equipment weights were taken as an average of three or more different types of each piece of machinery, drawn from a range of industry sources. Based on Glithero et al. (2012), the machinery is assumed to be made of steel and embedded energy and emissions per kg of steel are assumed to be 23 GJ kg^− 1^ and 1.56 kg CO_2_-eq kg^− 1^. The lifespan is assumed to be 3000 h.

Machine	Weight (kg)	Indirect energy (MJ h^− 1^)	Indirect emissions (kg CO_2_-eq h^− 1^)
One-pass cultivator (4.5 m)	7350	56.35	3.83
Medium disc harrow (2–3 m)	1720	13.19	0.90
Spring-tine harrow (6 m)	3500	26.83	1.81

**Table 4:** Pesticide costs and number of applications per crop. Calculated using the original calculations presented in Glithero et al. (2012) using prices from ABC (2014).

Crop	Pesticide category	No. of sprays	Original price (£ ha^− 1^)	New price (£ ha^− 1^)	Difference (%)
WW	Fungicides	3	68.95	77.93	+ 13.0
Herbicides	3	36.01	44.04	+ 22.3
Growth regulators	2	22.54	23.50	+ 4.3
Insecticides	1	5.80	4.96	− 14.5
Seed treatments and molluscicides^a^	1	14.19	14.81	+ 4.4
Seed treatments and molluscicides^b^	1	16.09	16.79	+ 4.3
WB	Fungicides	2	45.97	51.95	+ 13.0
Herbicides	2	24.01	29.36	+ 22.3
Growth regulators	1	11.27	11.75	+ 4.3
Insecticides	1	5.80	4.96	− 14.5
Seed treatments and molluscicides	1	13.72	14.32	+ 4.4
SB	Fungicides	2	45.97	51.95	+ 13.0
Herbicides	2	24.01	29.36	+ 22.3
Seed treatments and molluscicides	1	15.61	16.29	+ 4.4
WOSR	Fungicides	2	29.14	22.13	− 24.1
Herbicides	3	89.43	80.36	− 10.1
Insecticides	2	12.87	11.50	− 10.6
Seed treatments and molluscicides	2	20.66	24.50	+ 18.6
WFB	Fungicides	2	37.01	30.33	− 18.0
Herbicides	2	64.93	73.33	+ 12.9
Insecticides	2	12.87	13.25	+ 3.0

a For a first winter wheat.

b For a second or continuous winter wheat.

**Table 5:** Crop prices from the original model and new prices reflecting average crop prices from November 2013 to October 2014.

Crop	Original price (£ tonne^− 1^)	New price (£ tonne^− 1^)	Change (%)
WW (grain)^a^	172.36	144.56	− 16.1
WW (straw)^b^	43.00	43.50	+ 1.2
WB, SB (grain)^a^	164.42	122.64	− 25.4
WB, SB (straw)^b^	59.00	51.92	− 12.0
WOSR^c^	374.08	290.49	− 22.3
WFB^c^	206.67	221.30	+ 7.1

a Defra (UK weekly commodity prices, source HGCA).

b Defra commodity prices: Hay & Straw, England and Wales average prices.

c Selected feeding-stuffs prices, Great Britain. This data is available from Anon. (2014).

**Table 6:** Contractors' costs in the 2011 and 2014 MEETA models.

Machinery	2011 contract cost (£ hr.^− 1^)	2014 contract cost (£ hr.^− 1^)	Difference (%)
Small tractor	25.01	23.90	− 4.4
Medium tractor	35.81	35.28	− 1.5
Large tractor	50.21	44.61	− 11.2
Combine harvester	121.00	123.48	+ 2.0
Swather	57.00	57.87	+ 1.5
Baler (round bales)	45.63	53.51	+ 17.3

**Table 7:** Crop mixes and corresponding gross margins, net energy and GHG emissions for the three optimisation scenarios for each tillage system.

	Tillage system					
CT	RP	DRT	SRT1	SRT2	ZT
*Maximised gross margins*						
WW (SR, 75% N)	133.33	138.36	186.48	186.48	186.48	186.48
WB (ASR, SR)	133.33	61.64	27.04	27.04	27.04	27.04
WOSR	133.33	200.0	186.48	186.48	186.48	186.48
Gross margins (£ farm^− 1^)	285,782	326,522	351,366	351,508	355,175	357,717
Net energy (GJ farm^− 1^)	25,727	26,211	27,668	27,684	27,910	28,067
GHG emissions (kg CO_2_-eq farm^− 1^)	1,767,137	1,679,049	1,600,597	1,599,274	1,579,982	1,566,522

*Maximised net energy*						
WW (SR, 75% N)	200.00	200.00	200.00	200.00	200.00	200.00
WOSR	–	200.00	200.00	200.00	200.00	200.00
WFB	200.00	–	–	–	–	–
Gross margins (£ farm^− 1^)	268,172	320,719	347,016	336,757	350,902	353,440
Net energy (GJ farm^− 1^)	268,172	26,947	27,771	27,788	28,013	28,161
GHG emissions (kg CO_2_-eq farm^− 1^)	935,308	1,662,348	1,591,680	1,590,358	1,571,066	1,558,385

*Minimised GHG emissions*						
WOSR	–	200.00	–	–	–	–
WW (50% N)	200.00	200.00	200.00	200.00	200.00	200.00
WFB	200.00	–	200.00	200.00	200.00	200.00
Gross margins (£ farm^− 1^)	242,188	256,025	282,011	280,080	283,747	288,229
Net energy (GJ farm^− 1^)	20,937	21,060	22,007	21,892	22,117	22,402
GHG emissions (kg CO_2_-eq farm^− 1^)	764,305	753,759	672,540	682,424	663,132	638,920

Key: SR — straw removed; ASR — grown after the previous crop had straw removed; % N — percentage of nitrogenous fertiliser applied relative to recommended levels.

**Table 8:** Fuel, labour, machinery, and contractor costs and the resultant net margins for each tillage system for crop mixes under the gross margin maximisation objective.

	Tillage system					
CT	RP	DRT	SRT1	SRT2	ZT
GM (£ ha^− 1^)	714	816	879	888	878	894
Fuel use (L farm^− 1^)	91,951	71,193	48,515	48,294	42,605	38,661
Contractors' fees (£ farm^− 1^)	43,748	11,364	9689	9689	9689	9689
Machinery costs (£ farm^− 1^)	118,591	114,786	110,735	89,743	86,058	82,545
Machinery costs (£ ha^− 1^)	296	287	277	224	215	206
Fuel costs (£ farm^− 1^)	59,262	45,884	31,268	31,126	27,459	24,917
Fuel costs (£ ha^− 1^)	148	115	78	78	69	62
Labour costs (£ farm^− 1^)	27,432	23,484	16,644	16,536	18,804	14,964
Labour costs (£ ha^− 1^)	69	59	42	41	47	37
Net margins (£ farm^− 1^)	172,712	211,625	240,640	260,490	270,222	275,045
Net margins (£ ha^− 1^)	432	529	602	651	676	688

**Table 9:** Selected crop mix for maximised gross margins when all crops excluding spring barley incur a 20% yield penalty.

	Gross margin maximised
Crop mix	
WW (SR, 75% N)	133.33
WB (ASR, SR)	30.68
WOSR	133.33
SB	102.65
Gross margins (£ farm^− 1^)	254,247
Net Energy (GJ farm^− 1^)	21,868
GHG emissions (kg CO_2_-eq farm^− 1^)	1,483,700

Key: SR — straw removed; ASR — grown after the previous crop had straw removed; % N — percentage of nitrogenous fertiliser applied relative to recommended levels.

**Table 10:** Optimal crop mixes for maximising GMs and net energy, and minimising GHG emissions when taking account of *Greening* requirements.

	Tillage system	
CT	ZT
*Maximised gross margins*		
WW (SR, 75% N)	133.33	186.48
WB (ASR, SR)	133.33	27.04
WOSR	133.33	186.48
Gross margins (£ farm^− 1^)	285,782	357,717
Net energy (GJ farm^− 1^)	25,727	28,067
GHG emissions (kg CO_2_-eq farm^− 1^)	1,767,137	1,566,522

*Maximised net energy*		
WW (SR, 75% N)	200.00	190.00
WB (ASR, SR)	–	20.00
WOSR	20.00	190.00
WFB	180.00	–
Gross margins (£ farm^− 1^)	270,687	354,025
Net energy (GJ farm^− 1^)	26,142	28,122
GHG emissions (kg CO_2_-eq farm^− 1^)	101,633	1,561,771

*Minimised GHG emissions*		
WW(50% N)	180.00	180.00
WFB	200.00	200.00
SB	20.00	20.00
Gross margins (£ farm^− 1^)	238,683	281,251
Net energy (GJ farm^− 1^)	20,559	21,988
GHG emissions (kg CO_2_-eq farm^− 1^)	764,569	642,223

Key: SR — straw removed; ASR — grown after the previous crop had straw removed; % N — percentage of nitrogenous fertiliser applied relative to recommended levels.

**Table 11:** Optimised GMs, NE and GHG emissions for the different tillage systems for the 2014 price scenario with the ‘Greening’ requirement.

	Tillage system					
CT	RP	DRT	SRT1	SRT2	ZT
*Maximised gross margins*						
WW (SR, 75% N)	180.00	200.00	200.00	200.00	200.00	200.00
WB (ASR, SR)	20.00	–	–	–	–	–
WOSR	–	20.00	20.00	81.89	81.89	20.00
WFB	200.00	180.00	180.00	118.11	118.11	180.00
Gross margins (£ farm^− 1^)	233,006	245,692	270,501	268,858	272,524	276,716
Net energy (GJ farm^− 1^)	25,962	26,335	27,283	27,390	27,615	27,677
GHG emissions (kg CO_2_-eq farm^− 1^)	937,198	999,608	918,356	1,155,161	1,135,869	884,768

*Maximised net energy*						
WW (SR, 75% N)	200.00	200.00	190.00	190.00	190.00	190.00
WB (ASR, SR)	–	–	20.00	20.00	20.00	20.00
WOSR	20.00	180.00	190.00	190.00	190.00	190.00
WFB	180.00	20.00	–	–	–	–
Gross margins (£ farm^− 1^)	230,595	233,501	258,331	254,329	257,995	262,436
Net energy (GJ farm^− 1^)	26,142	26,766	27,726	27,742	27,967	28,122
GHG emissions (kg CO_2_-eq farm^− 1^)	1,016,233	1,598,378	1,595,643	1,594,321	1,575,029	1,561,771

*Minimised GHG emissions*						
WW (50% N)	180.00	180.00	180.00	180.00	180.00	180.00
WFB	200.00	200.00	200.00	200.00	200.00	200.00
SB	20.00	20.00	20.00	20.00	20.00	20.00
Gross margins (£ farm^− 1^)	204,298	216,816	238,475	236,545	240,211	244,691
Net energy (GJ farm^− 1^)	20,559	20,681	21,593	21,478	21,703	21,988
GHG emissions (kg CO_2_-eq farm^− 1^)	764,569	754,022	675,842	685,726	666,434	642,223

Key: SR — straw removed; ASR — grown after the previous crop had straw removed; % N — percentage of nitrogenous fertiliser applied relative to recommended levels.

## Footnotes

1 Practitioners use a variety of names for the non-inversion tillage system. In this study, reduced tillage is used to refer to any tillage system that does not employ inversion. For a more detailed look at various terminologies used in the literature, see Table 1 in Townsend et al. (2016).

2 The original model assumes that a precision drill is used; this has a slower work-rate than the cultivation drill often used for drilling crops in cereal rotations. However, substituting in a cultivation drill has very little impact on overall output values and, therefore, the precision drill was retained for this analysis.

3 When assuming the following nutritional calories in kcal per kg of grain: 3400 (WW), 3540 (WB, SB), 3410 (WFB) and 3969 (WOSR, assuming an oil yield of 44.9%). Values taken from USDA (2016).
